# Complete Genome Sequence of Colistin-Resistant Escherichia fergusonii Strain EFCF056

**DOI:** 10.1128/MRA.01571-19

**Published:** 2020-02-06

**Authors:** Biao Tang, Yifei Chen, Ling Zhang, Jiang Chang, Xiaodong Xia, Hua Yang

**Affiliations:** a Institute of Quality and Standard for Agro-products, Zhejiang Academy of Agricultural Sciences, Hangzhou, Zhejiang, China; b State Key Laboratory for Managing Biotic and Chemical Threats to the Quality and Safety of Agro-products, Zhejiang Academy of Agricultural Sciences, Hangzhou, Zhejiang, China; c College of Food Science and Engineering, Northwest Agriculture and Forestry University, Yangling, Shaanxi, China; University of Rochester School of Medicine and Dentistry

## Abstract

Here, we report the complete genome sequence of colistin-resistant Escherichia fergusonii strain EFCF056, isolated from chicken feces. This genome contains six plasmids, including a 204,246-bp plasmid harboring the colistin resistance gene *mcr-1*. These results will increase our understanding of plasmid-mediated *mcr-1* gene presence and transmission in E. fergusonii.

## ANNOUNCEMENT

Of the eight Escherichia species (1, 2), E. fergusonii and E. coli are easily confused during isolation due to their phenotypic and genotypic similarities (1, 3). *E. fergusonii* is pathogenic to both humans and animals (1, 3, 4, 5, 6) and has reportedly acquired multiple-drug resistance (1, 6). Since there is limited research on antimicrobial resistance in *E. fergusonii*, an isolate from chicken feces from Zhejiang Province, China, was examined for antimicrobial resistance. Its whole-genome sequence is described here.

A single colony from a previously cultured isolate was selected and inoculated into 10 ml of LB broth for genomic DNA extraction (QIAprep Spin miniprep kit; Qiagen, Germany). The purity and quantity of the extracted DNA were examined using a NanoDrop One UV-visible (UV-vis) spectrophotometer (Thermo Fisher Scientific, USA) and a Qubit 3.0 fluorometer (Invitrogen, USA), respectively. Libraries were prepared (SQK-LSK109 kit; Oxford Nanopore Technologies [ONT]) and sequenced using FLO-MIN106D R9.4 flow cell (ONT) technology on a GridION sequencer (ONT) and repeated on an Illumina HiSeq platform. All software systems were operated on their default settings. Guppy v3.2.4 (ONT) was used for base calling of raw fast5 data and removal of adapter sequences. An Illumina sequencing library was generated using a NEXTflex DNA sequencing kit (Bioo Scientific, USA). A total of 16,654,064 paired-end reads (2 × 150 bp) were checked for quality and trimmed with Trimmomatic v0.36. All low-quality (*Q* < 20) data were filtered out. The 140,754 nanopore reads (total of 3,427,444,055 nucleotides) were assembled *de novo* using Canu v1.7.11 (7), achieving an *N*_50_ value and mean read size of 33,077 bp and 24,350 bp, respectively. The assembly was circularized using Circulator v1.5.1 and corrected by Illumina reads (497-fold coverage) using Pilon v1.22 software (8). The completed assembly consisted of seven contigs with an *N*_50_ contig size of 4,576,669 bp. Two clear GC skew shift points were found around the start codon of *dnaA* on the chromosomal sequence.

As shown in Table 1, this genome comprises a chromosome of 4,576,669 bp (GC content, 49.86%) and six plasmids. The gene prediction and annotation of this genome (NCBI Prokaryotic Genome Annotation Pipeline) revealed 5,082 protein-coding sequences (CDSs), 82 tRNA genes, and 7 rRNA operons. No rRNA genes were found in the plasmids. The average nucleotide identity value of genome sequences of *E. fergusonii* strains EFCF056 (GenBank accession number CP040805) and ATCC 35469 (accession number NC_011740) (9) was 98.53%.

**TABLE 1:** Genome features of Escherichia fergusonii strain EFCF056^*a*^

Name	Contig length (bp)	GC content (%)	Topology
Chromosome	4,576,669	49.86	Circular
Plasmid pEF01	204,246	47.86	Circular
Plasmid pEF02	90,871	53.37	Circular
Plasmid pEF03	80,801	50.67	Circular
Plasmid pEF04	80,105	51.91	Circular
Plasmid pEF05	79,936	51.89	Circular
Plasmid pEF06	67,036	39.41	Circular

a Carrying antimicrobial resistance genes *bla*_TEM-1A_, *bla*_CTX-M-65_, *bla*_OXA-1_, *bla*_TEM-1B_, *bla*_CTX-M-55_, *aac(3)-IId*, *aph(3′)-Ia*, *aph(3″)-Ib*, *aph(6)-Id*, *rmtB*, *aac(6')-Ib-cr*, *aadA2, mcr-1*, *qnrS2*, *aac(6')-Ib-cr*, *oqxA*, *oqxB*, *fosA, mph*(A), *floR*, *catA1*, *catB3, ARR*-3, *sul1*, *sul2*, *dfrA12*, and *tet*(A).

The acquired antimicrobial resistance genes were predicted via the ResFinder database (10). In addition, there were *aac(3)-IId*, *aadA2*, *aph(3″)-Ib*, *aph(6)-Id*, *aph(3′)-Ia*, *bla*_TEM-1B_, *mph*(A), *catA1*, *sul1*, *sul2*, *tet*(A), and *dfrA12* genes in plasmid pEF01 carrying the *mcr-1* gene. Using the broth microdilution method, the EFCF056 isolate was found to be resistant to 11 agents, including colistin (MIC, 4 mg/liter).

The results showed that *E. fergusonii* might be a reservoir for antimicrobial resistance genes. Further studies are required to investigate the MIC distribution and frequency and the importance of the colistin resistance gene *mcr-1*.

### Data availability.

The complete genome sequence of Escherichia fergusonii EFCF056 has been deposited in GenBank/ENA/DDBJ under accession numbers CP040805, CP040806, CP040807, CP040808, CP040809, CP040810, and CP040811. The accession numbers of the original read data set in the SRA are SRR9214441 (Nanopore), SRR10686670 (Nanopore), and SRR10718121 (Illumina).
